# Discrepancies in Subjective Perceptions of Hydrocephalus Management and Self-Reported Outcomes

**DOI:** 10.3390/jcm13237205

**Published:** 2024-11-27

**Authors:** Julian Zipfel, Zoltan Filip, Cristina Kohlmann-Dell’Acqua, Susan Noell, Leonidas Trakolis

**Affiliations:** 1 Department of Neurotechnology and Neurosurgery, University Hospital Tuebingen, 72076 Tübingen, Germany; cristina.kohlmann-dellacqua@med.uni-tuebingen.de (C.K.-D.); leonidastra86@hotmail.com (L.T.); 2 Centre for Clinical Studies, University Hospital Tuebingen, 72076 Tübingen, Germany; 3 Neuropsychiatric Study Centre, University Hospital Tuebingen, 72076 Tübingen, Germany; 4 Department of Neurosurgery, St. Luke Hospital, 552 36 Thessaloniki, Greece

**Keywords:** hydrocephalus, quality of life, self-reported outcomes

## Abstract

**Background/Objectives**: Despite surgical interventions with advances in endoscopic procedures as well as shunt technologies, the quality of life in patients with hydrocephalus can be poor. Clinical experience suggests discrepancies between objective measures of treatment success and subjective patient satisfaction. With this study, we retrospectively investigated patients’ knowledge of their treatment as well as their satisfaction with received interventions. **Methods**: Retrospective analysis of self-reporting forms, routinely handed out in the hydrocephalus clinic of a tertiary neurosurgical center, was performed. Clinical data were gathered between 1 January 2020 and 31 March 2023. Correlation of self-reporting forms and available clinical data was performed. **Results**: A total of 261 forms from 215 patients were obtained. The mean age at visit was 57.5 ± 18.5 years (range 19–88). The most common pathology was normal pressure hydrocephalus (NPH, 31.6%); 31.2% had an occlusive etiology, 22.3% posthemorrhagic, 9.8% benign intracranial hypertension and 5.1% another pathology. Overall, 53% of patients (*n* = 114) indicated the correct therapy on the self-reporting forms (χ^2^ (56) = 100.986, *p* < 0.001). Symptoms and subjective benefit did not differ in the different types of provided therapy. **Conclusions**: Merely half of the patients with hydrocephalus are able to correctly indicate the treatment they had received. The type of shunt valve did not affect the rate of self-reported symptoms. The symptoms and subjective benefits did not differ in the different types of provided therapy. Poor patient knowledge could correlate with poor self-reported quality of life. Medical professionals should emphasize and advocate for better patient education.

## 1. Introduction

Hydrocephalus is one of the most common pathologies in neurosurgical practice with significant morbidity and socioeconomic impact. Despite advancements in surgical interventions, including endoscopic procedures and shunt technologies, the quality of life for patients with hydrocephalus can often be poor [1]. Clinical experience in treating patients with hydrocephalus suggests discrepancies between objective measures of treatment success and subjective patient satisfaction. Improving or maintaining quality of life is one of the most important goals of hydrocephalus treatment and may be more challenging due to the life-long persistence of the pathology. For instance, patients with normal pressure hydrocephalus (NPH) report significant impairments in their quality of life after treatment [2]. Improvement of gait and incontinence are validated objective markers of treatment response [3,4,5]. Conversely, other forms of hydrocephalus usually are not associated with these symptoms, and in younger patients who undergo shunt implantation, up to 81% may require shunt revision at some point in their lives, often due to shunt dysfunction or infection [6].

Determining the success of treatment can thus be challenging, as medical perspectives may not always align with those of the patients. Similar difficulties arise in cases of post-traumatic hydrocephalus, where the patients’ general condition may complicate the definition of “treatment success” [7]. In patients with NPH, the self-reported improvement after shunt surgery is significant, especially concerning the gait disturbance [1]. Evaluation of this subgroup of patients is challenging due to differences in cognitive performance when compared to other hydrocephalus types [8].

The first year after shunt implantation can set the course for the subsequent quality of life [9], as most shunt complications or therapy failures are encountered during this period. Post-surgical complications are a reality for many patients with hydrocephalus and can significantly reduce their quality of life over time. Around two-thirds of patients remain shunt-failure-free the first year and less than half the second year [10]. Some patients may be unaware of their specific disorder and the new realities they face after shunt treatment. Poor patient education can lead to hypersensitivity and psychosomatic disorders, particularly in children [11]. Longitudinal data suggest that subjectively perceived quality of life may decrease from childhood to adulthood compared to healthy controls [12]. Additional comorbidities may further diminish patients’ quality of life, independent of hydrocephalus.

Currently, no clinical standard exists for assessing the quality of life in patients with hydrocephalus, although various questionnaires have been proposed [13]. This is what led us to the design of this study on the first place. However, self-reported studies are challenging in this patient population, as hydrocephalus is often accompanied by reduced cognitive ability. To solve this problem, caregivers and patients’ parents (in the case of child hydrocephalus) were recruited for previous studies [6] In our case, we report only about adult hydrocephalus patients so that the help of caregivers was in some cases necessary.

In our clinic, we follow hydrocephalus patients with all etiologies and around 600 visits per year. In this study, we retrospectively investigated patients’ knowledge of their treatment as well as their satisfaction with the interventions received. Specifically, self-reported outcomes, symptoms and therapies were correlated with the underlying pathology, documented treatment and objective outcome measures.

## 2. Materials and Methods

We retrospectively analyzed all available hydrocephalus clinic self-reporting forms between 1 January 2020 and 31 March 2023. During this time, these forms were routinely handed out in our hydrocephalus clinic during waiting time. Completion was voluntary. The forms were then collected by the respective neurosurgeon upon patient contact in the exam room. Over the observational period, the team working on the project and collecting the data remained constant. Conceptualization of the form was performed by the senior authors. The questionnaire can be found in the Supplementary Material. It included general questions about the patients’ understanding of their disease and treatment, as well as current symptoms, some general questions about life circumstances, a visual analogue scale of wellbeing and the Herth Hope Scale (HHS) [14,15]. From the twelve individual scales of the HHS, a sum score was formed. Since a score between one and four could be assigned for each question, the possible range of this sum score was between 12 and 48.

Completion of the form had to be carried out by either the patient or their legal guardian. Inclusion criteria were as follows: age of 18 years or older, completion of the form by the patient or legal guardian, consent to data collection. Exclusion criteria were as follows: insufficient data points, inability to consent or refusal of data collection. Only those forms returned during the hydrocephalus clinic visit were used. No case was excluded.

All available forms were included, and additional data points were taken from patients’ charts (type of therapy, type of shunt valve, reoperations, revisions, medical history). One senior researcher performed the data collection.

Statistics were analyzed using SPSS Statistics 29 (IBM, Armonk, NY, USA). Continuous data were presented as mean (±SD), whereas categorical data were shown as percentages. For the comparison of parameters between two groups (e.g., male vs. female), the *t*-test for independent samples was applied, but only when the data were interval-scaled and it could be assumed that they followed a normal distribution. If normality could not be assumed, a test for non-parametric data (Mann–Whitney U test) was used for the comparison between two groups (e.g., male vs. female). Paired samples (parameters at time point 1 vs. parameters at time point 2) were analyzed using the Wilcoxon test (non-parametric). A parametric method such as the *t*-test for paired samples was not applicable here, as the corresponding data were all interval-scaled (e.g., VAS scale or ranking score). Simple comparisons of numbers were performed using the Chi-squared test. *p* values < 0.05 were regarded as significant. This study has been approved by the local ethics committee and a waiver was granted for individual patient consent (630/2021BO2, 11 October 2021).

## 3. Results

### 3.1. Basic Patient Information

In total, 261 forms were analyzed corresponding to the same number of clinic visits in 215 patients. Most patients completed only one form during the observation period, 28/215 returned for a second visit, 6 for two more visits and 2 patients returned for three more visits and completed the forms. The mean number of total visits to our clinic was 12.6 ± 10.2 (range 1–62).

**Figure 1:**
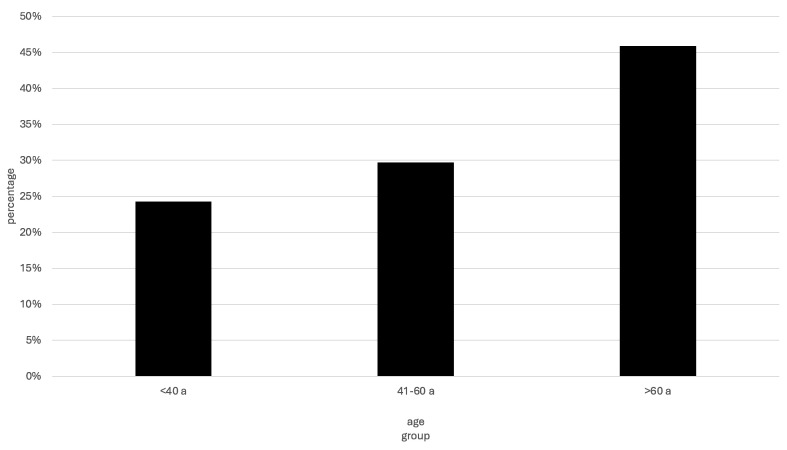
Distribution of patients according to age group.

The mean age at the respective visits was 57.5 ± 18.5 years (range 19–88). The age distribution is shown in Figure 1.

**Figure 2:**
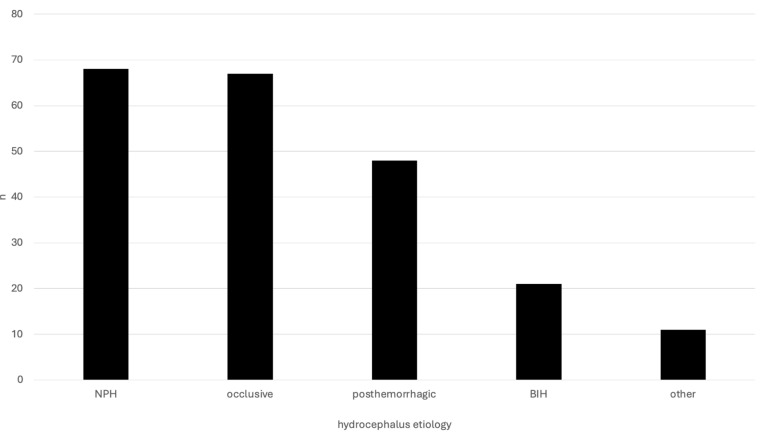
Number of patients according to hydrocephalus etiology.

Of all of the patients, 52.6% were female (*n* = 113) and 47.4% male (*n* = 102). The distribution of underlying hydrocephalus pathologies was as follows: 31.6% NPH (*n* = 68), 31.2% occlusive (*n* = 67), 22.3% posthemorrhagic (*n* = 48), 9.8% benign intracranial hypertension (BIH; *n* = 21), 5.1% other (*n* = 11) (see Figure 2).

### 3.2. Self-Reporting Forms

The self-reported symptoms at the time of clinic visit were as follows: headaches 55.6% (*n* = 120), incontinence 66.5% (*n* = 143), gait ataxia 48.8% (*n* = 105), nausea 83.7% (*n* = 180), concentration deficit 54.6% (*n* = 113), vertigo 54.4% (*n* = 117), dementia 90.7% (*n* = 195) and visual deficit 24.7% (*n* = 53).

Self-reported therapy was distributed as follows: 40.5% none (*n* = 87), 44.2% VP shunt (*n* = 95), 3.7% VA shunt (*n* = 8), 1.9% cerebral stent (*n* = 4), 0.9% LA shunt (*n* = 2), 0.9% ETV (*n* = 2), 1.9% LP shunt (*n* = 4), 3.3% pharmacological (*n* = 7), 2.8% missing (*n* = 6).

The actual received therapies as documented in the patients’ charts were as follows: 11.6% none (*n* = 25), 75.3% VP shunt (*n* = 162), 2.8% VA shunt (*n* = 6), 0.5% cerebral stent (*n* = 1), 2.3% ETV (*n* = 5), 4.7% LP shunt (*n* = 10), 0.5% pharmacological (*n* = 1), 0.5% cystoperitoneal shunt (*n* = 1), 1.9% missing (*n* = 4).

Overall, 53% of the patients (*n* = 114) indicated the correct therapy on the self-reporting forms (χ^2^ (56) = 100.986, *p* < 0.001). The rate of incorrect reports of therapy was tendentially higher in patients with self-reported mnestic deficit, urinary incontinence and dementia. Patients without therapy had tendentially but insignificantly higher rates of correct indication on the form (68.0%, 17/25); in patients with VP shunts, only 86/162 gave a correct answer (54.8%, *p* = 0.608).

The mean visual analogue scale (VAS) for overall well-being was 4.3 ± 2.1 with a range of 0–10. Of all of the patients, 89.2% documented feeling well informed about their disease and the therapy. A subjective benefit from surgery was documented by 73.0%.

### 3.3. Comparison of Diagnoses

**Figure 3:**
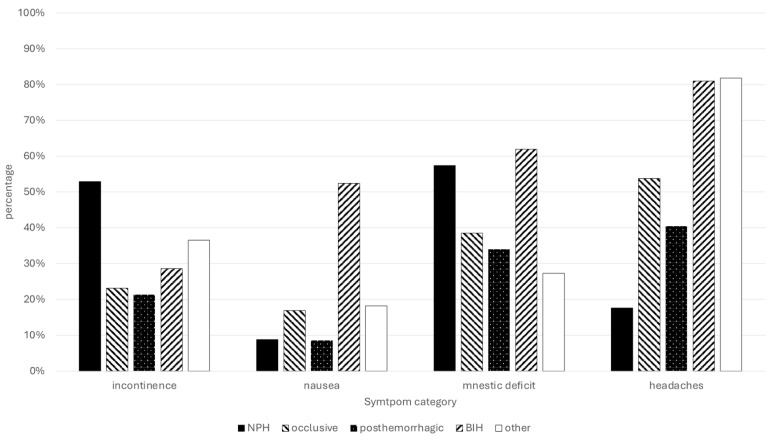
Percentage of self-reported symptoms according to hydrocephalus pathology.

The rate of self-reported incontinence was significantly higher in NPH as compared to other diagnoses (occlusive 23.1%, posthemorrhagic 21.3%, NPH 52.9%, BIH 28.6% and others 36.5%; overall, 33.5%; χ^2^(4) = 18.31, *p* = 0.001, ϕ = 0.292). The same was true for self-reported gait ataxia (occlusive 41.5%, posthemorrhagic 34.0%, NPH 80.9%, BIH 23.8%, other 45.5%; overall, 50.9%; χ^2^(4) = 38.38, *p* < 0.001, ϕ = 0.42). Nausea was reported significantly more often in patients with BIH (occlusive 16.9%, posthemorrhagic 8.5%, NPH 8.8%, BIH 52.4%, other 18.2%, χ^2^(4) = 25.28, *p* < 0.001, ϕ = 0.345). Mnestic deficits were reported more often in NPH and BIH (occlusive 38.5%, posthemorrhagic 34.0%, NPH 57.4%, BIH 61.9%, other 27.3%; overall, 45.3%; χ^2^(4) = 11.397, *p* = 0.022, ϕ = 0.232). Headaches were reported more often in BIH and other diagnoses (occlusive 53.8%, posthemorrhagic 40.4%, NPH 17.6%, BIH 81.0%, other 81.8%; overall, 43.4%; χ^2^(4) = 11.397, *p* = 0.022, ϕ = 0.232) (see Figure 3).

Self-reported impairment of focus (*p* = 0.050), vertigo (*p* = 0.067) and subjective benefit (*p* = 0.073) did not differ significantly between diagnosis groups.

### 3.4. Comparison of Therapy

The type of shunt valve did not affect the rate of self-reported headaches, gait ataxia, nausea, concentration deficit, vertigo, mnestic deficit or benefit from therapy. Symptoms and subjective benefit did not differ in the different types of provided therapy.

### 3.5. Comparison of First vs. Last Visit

For 37/215 patients, more than one clinic form was available (2 in 59.5% (*n* = 22), 3 in 40.5% (*n* = 15)). No significant changes were observed in the provided answers. Comparison revealed the following results: headaches remained unchanged (54.1% *n* = 20), incontinence was unchanged (43.2% *n* = 16 vs. 45.9% *n* = 17), gait ataxia tendentially increased (45.9% *n* = 17 vs. 51.4% *n* = 19), nausea tendentially increased (16.2% *n* = 6 vs. 21.6% *n* = 8) and vertigo tendentially increased (43.2% *n* = 16 vs. 51.4% *n* = 19), as well as mnestic deficits (54.1% *n* = 20 vs. 59.5% *n* = 22) and visual deficits (18.9% *n* = 7 vs. 27% *n* = 10).

The median difference between the visit at which a form was completed for the first time (initial examination) and the visit during which the last examination (with form) was carried out was 10 months (9.7 ± 12.5 months). Table 1, Table 2 and Table 3 give an overview of the comparisons between the first and last test.

From the twelve individual scales of the questions about the personal situation (attitude to life), a sum score was formed. A score between 1 and 4 meant a possible range of this sum score was between 12 and 48. For the initial examination, this resulted in an average score of 40.9 ± 6.1 (median 43.0 with a range of 26 to 48). In the final survey, the mean score of 40.2 ± 6.7 was at a comparable level (median 42.0 with a range of 24 to 48). The difference between T1 and T2 was not statistically significant (*p* = 0.815 in the Wilcoxon test).

## 4. Discussion

With the presented study, we showed that self-reporting forms can be a valuable addition in the clinical routine to identify the concerns of patients. On the one hand, more than 80% of the patients documented feeling well informed about their disease and treatment. Conversely, half of the patients failed to recognize the correct therapy they received, particularly those who underwent VPS implantation.

Hydrocephalus can occur at any time in a patient’s life and is often associated with relevant morbidity. The individual level of knowledge can pose a significant challenge for clinicians to explain the condition thoroughly yet simply enough for the patient to understand. Some patients have additional cognitive deficiencies, especially in NPH, while others may be overwhelmed when the diagnosis is in the context of or following another severe issue. Neuropsychological impairments in patients with hydrocephalus are complex and individual [16]. The pathophysiology of hydrocephalus is complex, requiring considerable experience from clinicians to treat effectively [17].

Consequently, explaining these complex matters to patients in a comprehensible manner is even more challenging. Medical terminology should be avoided when possible, and knowledge must be broken down into simple terms. Specifically adapted education material may be of assistance [18]. Simple language, patience and redundancy may be necessary. If patients do not recognize the therapy they underwent, we cannot expect them to adhere to recommendations. Even for medical experts, the understanding of the pathophysiology of hydrocephalus incomplete [4].

Poor patient education and cognitive impairments may explain one of the most significant results of our questionnaire: many patients were relatively unaware of the therapy they had undergone. This observation was made despite the fact that patients who undergo surgery in our center are thoroughly informed about the type and risks of the procedure. Written consent is mandatory after the patient has heard, read and understood the planned therapy, and the presence of close relatives during the consultation is always encouraged, particularly for patients with cognitive impairment. Nevertheless, one-third of the patients were unsure or unaware of the procedure or therapy they had received. Furthermore, all of the patients in our cohort received copies of clinic notes and consent forms with detailed information, and those with a ventriculoperitoneal shunt (VPS) received a shunt passport detailing the type of valve and current configuration.

Despite the fact that many studies have reported on the success rates of hydrocephalus treatment, very few dealt with the quality of life of those patients and even fewer are based on self-reports. Kulkarni et al. developed a Hydrocephalus Outcome Questionnaire (HOQ) for children with hydrocephalus in order to have a quantitative health status measure [6]. This self-reporting questionnaire was one of the first attempts to enlighten the effect of hydrocephalus treatment from the patients’ point of view (or in this case from their parents’). Our experience with adult patients showed that their satisfaction is directly analogous to their understanding of the disease and the treatment performed. In our presented study, we were able to identify a discrepancy between 89% of the patients feeling well informed but 53% being able to accurately indicate the treatment they received on self-reporting forms.

Hydrocephalus is a complex disorder that requires individualized treatment. Various factors must be considered, including socio-economic status, age and general condition, which significantly impact the understanding of the disease—not only for pediatric patients [19,20] but also for adults [21,22]. Due to the complexity of the symptoms (which include both somatic and psychological manifestations), these patients should be treated with extra care. More time and patience should be offered during each consultation, and an interdisciplinary team involving neurologists and psychologists should be considered in some cases. The presence of close relatives, social workers or caregivers during consultations should also be encouraged. Additionally, patient education should be prioritized. Various methods for educating patients have been proposed, such as support groups and online videos, which have shown promising results [23,24]. This could prove beneficial for treating hospitals and clinicians by reducing unnecessary patient visits. Notably, the mean number of total patient visits to our clinic was 12.6 ± 10.2 (range 1–62).

To effectively educate patients, their knowledge should first be assessed. Depending on their socio-economic background, patients’ understandings of basic medical information can vary significantly, necessitating different educational strategies for each case. Self-reporting forms, like the one we used, could be useful tools in this regard. Prospective assessment of patients’ knowledge about their disease and the available therapies could profoundly improve their outcomes. If patients understand their problems and potential solutions without holding unrealistic expectations, they are likely to be more compliant and satisfied in the future. Furthermore, support groups may help educate patients [18].

Adults with hydrocephalus acquired during infancy showed similar results to healthy individuals regarding mental health and social functioning [25]. This highlights the need to distinguish these patients from those who develop hydrocephalus as adults. There is a general lack of data concerning the quality of life for the adult population over time. Tisell et al. examined a cohort of 109 patients and assessed their sense of well-being as well as changes in common hydrocephalus symptoms [24]. In line with our results, half of these patients reported feeling better over time, while only one-third remained functionally improved.

NPH patients are a particular category, as these patients are more commonly cognitively impaired as compared to other etiologies of hydrocephalus. Difficulties in focus, memory and attention as well as psychomotor deceleration complete their cognitive profile. Often, this profile will be enriched with overlapping comorbidities, such as neurodegenerative diseases [26] or senile urinary incontinence. Consequently, the patient satisfaction may be influenced by pre-existing comorbidities [27]. An extended (60 months) follow-up study showed and emphasized the negative development of some patients due to aging and comorbidities but also the importance of weight and pre-operative cognitive status [28].

In general, shunt surgery in patients with NPH is associated with increased aspects of social life [27,29,30,31]. Unsurprisingly, typical NPH symptoms, such as urinary incontinence and gait ataxia, were self-reported more frequently by patients with this diagnosis compared to others, while nausea and headaches were reported more often in patients with benign intracranial hypertension (BIH). This suggests that patients, regardless of the type of hydrocephalus they have, can identify and report their symptoms. Interestingly, half of the patients failed to recognize the correct therapy they received, particularly those who underwent VPS implantation. In cases of shunt implantation as therapy, the type of valve did not affect the rate of self-reported symptoms such as headaches, gait ataxia, nausea, concentration deficits, vertigo or mnestic deficits, nor did it influence the perceived benefit from therapy. Although more dramatic improvements have been reported in shunt patients, symptoms and subjective benefits did not significantly differ across the various types of provided therapy [1].

Limitations of our study include the relatively short period of three years during which we collected forms and thus a limited number of patients, attributed to changes within our hydrocephalus team. We believe that the treating clinician plays a crucial role in patient education and evaluation, so we opted to maintain a smaller but more homogeneous cohort. Another limitation is that the form we used is custom-made by our team, making it challenging to directly compare our results with those from other clinics. A prospective multicentric study could further illuminate this matter.

## 5. Conclusions

Many patients under neurosurgical care for hydrocephalus or BIH show discrepancies in subjective perceptions of hydrocephalus management and self-reported outcomes. There is a large knowledge and educational gap which needs to be brought to the attention of neurosurgeons caring for their patients. Overall, medical professionals should always consider and advocate for simple language when talking to patients and their close ones. To improve patient education, information material and “up-to-date” shunt passports for patients should be considered.

## Figures and Tables

**Table 1:** Symptoms between first and last test.

	1st Test		2nd Test	
	*n*	%	*n*	%
Headaches	20	54.1	20	54.1
Urinary incontinence	16	43.2	17	45.9
Ataxia	17	45.9	19	51.4
Nausea	6	16.2	8	21.6
Concentration deficit	19	51.4	19	51.4
Vertigo	16	43.2	19	51.4
Mnestic deficit	20	54.1	22	59.5
Dementia	4	10.8	8	21.6
Visual deficit	7	18.9	10	27.0

**Table 2:** Changes in symptoms between first and last test.

	Unchanged		Newly Developed		Stopped	
	*n*	%	*n*	%	*n*	%
Headaches	33	89.2	2	5.4	2	5.4
Urinary incontinence	30	81.1	4	10.8	3	8.1
Ataxia	33	89.2	3	8.1	1	2.7
Nausea	33	89.2	3	8.1	1	2.7
Concentration deficit	31	83.8	3	8.1	3	8.1
Vertigo	32	86.5	4	10.8	2	5.4
Mnestic deficit	31	83.6	4	10.8	2	5.4
Dementia	33	89.2	4	10.8	0	0
Visual deficit	28	75.7	6	16.2	3	8.2

**Table 3:** Subjective level of information.

	1st Test		2nd Test	
Well-informed	*n*	%	*n*	%
No	3	8.1	2	5.4
Yes	30	81.1	33	89.2
N/A	4	10.8	2	5.4
Sum	37	100	37	100

## Data Availability

Data are available from the corresponding author upon reasonable request.

## Supplementary Materials

The following supporting information can be downloaded at: https://www.mdpi.com/article/10.3390/jcm13237205/s1.
