# Supplementary material for: Discrepancies in Subjective Perceptions of Hydrocephalus Management and Self-Reported Outcomes
Source: J Clin Med. 2024 Nov 27;13(23):7205. doi: 10.3390/jcm13237205 (PMC11642199; doi:10.3390/jcm13237205)

Universitätsklinikum Tübingen


Klinik für Neurochirurgie
Ärztlicher Direktor:
Prof. Dr. med. Marcos Tatagiba
Hoppe-Seyler-Straße 3
72076 Tübingen
Pforte: 07071/29-0


Klinik für Neurochirurgie • Hoppe-Seyler-Straße 3 • 72076 Tübingen

**Fragebogen Hydrocephalus**

Patientendaten

**Datum: 13.12.2019**

**Ist dies Ihr Erstbesuch bei uns?**

- ☐ ja
- ☐ nein

**Liegt bei Ihnen eine Behandlung mit eines/mehreren der folgenden vor?**

- ☐ ventrikuloperitonealer (VP) Shunt
- ☐ ventrikuloatrialer (VA) Shunt
- ☐ cerebraler Stent
- ☐ lumboatrialer (LA) Shunt
- ☐ Endoskopische Drittventrikulostromie (ETV)
- ☐ lumboperitonealer (LP) Shunt
- ☐ Medikamentöse Hirndrucktherapie (Acetazolamid, Furosemid,…)

**Liegt/Liegen bei Ihnen eine/mehrere der folgenden Störungen vor?**

- ☐ Kopfschmerzen
- ☐ Harninkontinenz
- ☐ Gangstörung
- ☐ Übelkeit
- ☐ Konzentrationsstörung
- ☐ Schwindel
- ☐ Gedächtnisstörung
- ☐ Demenzerkrankung
- ☐ Sehstörung, wenn ja: ☐ Doppelbilder ☐ Visusminderung ☐ Gesichtsfelddefizit
- ☐ Bauch-Operation (exkl. Shunt)

**Sind Sie aktuell berufstätig?**

- ☐ ja
- ☐ nein, ☐ aktuell krankgeschrieben

**Sind Sie aktuell in psychologischer Betreuung?**

- ☐ ja
- ☐ nein

**Denken Sie, dass eine psychologische Betreuung in Ihrer Situation hilfreich wäre?**

- ☐ ja
- ☐ nein

**Bitte beantworten Sie die folgenden Fragen zu Ihrer aktuellen persönlichen Situation!**

| | Trifft nie zu | Trifft selten zu | Trifft manch-mal zu | Trifft häufig zu |
|---|---|---|---|---|
| Ich habe eine positive Einstellung zum Leben | | | | |
| Ich habe kurzfristige und/oder langfristige Ziele | | | | |
| Ich fühle mich ganz allein | | | | |
| Ich kann auch in einer schwierigen Lage die Möglichkeiten sehen | | | | |
| Ich habe einen Glauben oder ein inneres Vertrauen, die mir Trost geben | | | | |
| Ich sehe ängstlich in die Zukunft | | | | |
| Ich kann mir glückliche Zeiten ins Gedächtnis rufen | | | | |
| Ich habe eine tiefe innere Kraft | | | | |
| Ich kann Fürsorge/Liebe geben und annehmen | | | | |
| Ich weiß meist, welchen Weg ich gehen möchte | | | | |
| Ich glaube, dass jeder Tag neue Möglichkeiten bietet | | | | |
| Ich empfinde mein Leben als wertvoll und lebenswert | | | | |

**Geht es Ihnen heute – im Vergleich zum letzten Besuch bei uns:**

☐ unverändert

☐ besser

☐ schlechter

**Fühlen Sie sich in Bezug auf Ihre Erkankung und Behandlung gut informiert?**

☐ ja

☐ nein

**Haben Sie von der letzten Operation profitiert?**

☐ ja

☐ nein

**Wie fühlen Sie sich heute?**

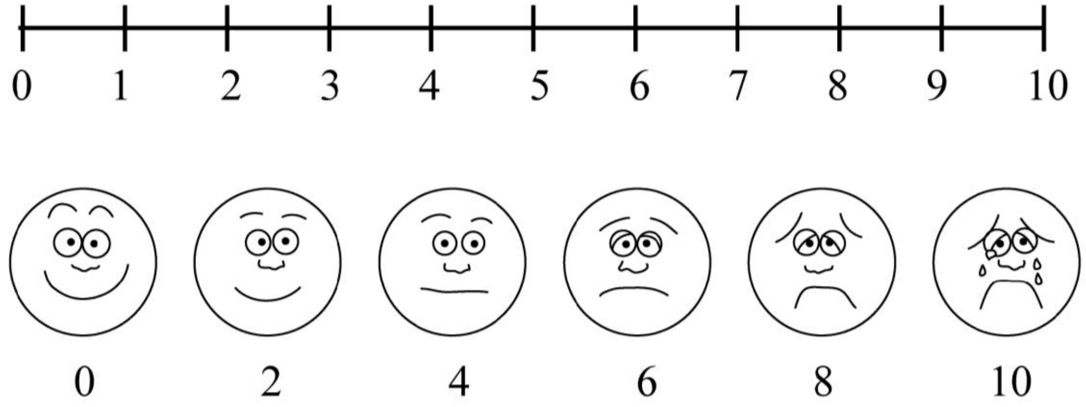

Supplement: Supplementary file 1 [file jcm-13-07205-s001.zip › jcm-3292163-supplementary.pdf]
